# Canada Source Watershed Polygons (Can-SWaP): A dataset for the protection of Canada’s municipal water supply

**DOI:** 10.1038/s41597-023-02732-9

**Published:** 2023-11-16

**Authors:** François-Nicolas Robinne, Chloé Lamache, Daniel K. Thompson, Jason A. Leach, Kevin D. Bladon

**Affiliations:** 1 Natural Resources Canada, Canadian Forest Service, 1219 Queen Street East, Sault Ste, Marie, ON P6A 2E5 Canada; 2 Pacific Salmon Foundation, Salmon Watershed Program, 320 – 1385 W 8th Ave, Vancouver, BC V6H 3V9 Canada; 3 College of Forestry, Oregon State University, 244 Peavy Forest Science Center, Corvallis, OR 97331-5704 USA

**Keywords:** Hydrology, Natural hazards, Geography

## Abstract

Over 80% of municipal (i.e., excluding industrial and agricultural) water use in Canada comes from streams, lakes, and reservoirs. These freshwater bodies and their catchments require adequate protection to secure drinking water supply for Canadians. Canada, like most countries, lacks a consolidated national dataset of municipal catchments, arguably due to gaps in data availability. Against this backdrop, we present the Canada Source Watershed Polygons dataset, or Can-SWaP. Can-SWaP was created using point locations of more than 3,300 municipal water licences defining rights to surface water withdrawal. Where possible, the resulting 1,574 catchments were assessed for accuracy in spatial coverage against provincial and local datasets. Each watershed in Can-SWaP has an estimated water volume used for municipal water purposes derived from licencing data, and several variables from RiverATLAS for investigating the integrity of surface drinking water sources in Canada. Furthermore, basing our method on the HydroSHEDS suite of global products offers a robust framework for the production of other national datasets following an established international standard.

## Background & Summary

The protection of drinking water in Canada follows the principles of the Integrated Water Resources Management (IWRM) paradigm^1^. Under the IWRM paradigm, water supply protection is typically approached through multiple spatial scales as part of a complex mix of competing interests within a river basin^1,2^. The *Water for Life* strategy, developed in Alberta, Canada, exemplifies this approach, with drinking water protection needs embedded within larger regional drainages and regulated through various programs and legal frameworks^3–5^. While planning for IWRM requires information on water sources, numerous historical and emerging drinking water issues across the country suggest that this information is likely incomplete. For example, the public-health impacts of contaminated water supply in Walkerton (Ontario)^6^ and North Battleford (Saskatchewan)^7^ remain vivid in national memory, while many Indigenous communities still suffer from long-term water advisories and their associated health effects^8,9^. Threats to water quality from heavy metals, over-allocation of water supplies, increasing forest disturbances, and climate change are only a few examples of water security concerns that exist in Canada nowadays^8,10–15^.

Since 2002, Canada’s source water protection has supposedly been achieved through the multi-barrier approach to safe drinking water^16–18^. According to this approach, source water characterization associated with the development of a source water protection (SWP) plan is the first step towards a level of protection “from source to tap” through multiple barriers implemented within the drinking water life cycle. In other words, a SWP plan should be concerned with the health of upstream areas supplying raw water—a hydrologic ecosystem service—to a drinking water treatment plant, as well as disturbances to this source that might pose water treatment challenges^4,19–21^. Although Canadian Provinces and Territories have each developed a legal framework coupled to technical guidelines for the elaboration of SWP plans, the fragmented water governance of Canada has contributed to a lack of coordinated data on the spatial characterization of source areas and, thus, a lack of drinking water protection across the country^5,10,22,23^. The absence of a national reference dataset of source watersheds is especially telling: while source water protection is supposed to start by delineating the watershed^18^, and while many jurisdictions are now committed to open government practises^24^, the fact that only three provinces have source catchment spatial data available in open access suggests more efforts are needed towards mapping and sharing source areas^25–27^. In a country where over 80% of domestic water needs come from surface water^28^, this is a significant data gap, especially given Canada’s engagement to reach—and maintain—Sustainable Development Goal No. 6, in particular No. 6.1 on achieving “universal and equitable access to safe and affordable drinking water for all”^29,30^.

Systemic inabilities to collect and share freshwater data have been a central problem to drinking water security in Canada, and while drinking water management is a Provincial or Territorial jurisdiction, growing pressures on municipal water supply all across the country support the development of a centralised, standardised, seamless, and open pan-Canadian dataset of source water catchments^10,25–27,31–33^. Spatial datasets are at the core of water management efforts^32^, and such national datasets would certainly contribute to drinking water security on many levels: getting a general picture of source water areas and their geography;^34,35^ helping with the “capacity gap” due to financial and staff limitations for creating these catchments in small communities;^36,37^ identifying nation-wide threats to drinking water sources (e.g., from climate change, disturbances, and land cover change^15,38–40^); and, finally, identifying further data gaps and designing data collection strategies (e.g., by modelling best coverage for water quality gauges)^25,41^. By describing the challenges met while building this dataset, we also hope to trigger important discussions regarding ways to standardise the structure of water licencing data and to streamline water data sharing between jurisdictions in Canada. We believe that the challenges we faced while assembling this dataset would be true in many countries, and we hope our experience holds useful lessons for people dealing with source water protection across the world.

Creating a drinking water catchment layer implies knowing the location of water intakes (i.e., devices/constructions within water bodies used to withdraw water). One might argue that using the location of drinking water treatment plants would be a sufficient proxy; there is, however, two major issues with this approach: (1) there is no readily available database locating drinking water plants in Canada, and, (2) several communities use water located outside of the community’s catchment but treated within the community (e.g., Winnipeg using Shoal Lake). The alternative option is to use the location of municipal water licences or permits. Using licences is advantageous for several reasons: (1) provinces and territories keep databases of valid licences, often in open access and spatial formats, or at least with the necessary information to create a spatial point dataset (i.e., coordinates; Table 1); (2) a licence usually corresponds to the location of a water intake—also called point of diversion—and thus allows the user to use these points as outlets for the creation of source catchments; an intake can host, however, multiple licences; and (3) even though municipal water use encompasses more than just drinking water production (e.g., firefighting, irrigation for small-scale farming and golf courses), it is fair to assume that most of the volume allocated through municipal licences will go towards domestic needs, since large industrial and agricultural users usually have their own water rights or even their own water intake.

**Table 1:** Municipal water licences and where to find them.

Province/Territory/Federal	Terminology	Data access	Data link
Alberta	Surface Water Diversion - Authorizations	Open (spatial)	https://geospatial.alberta.ca/titan/rest/services/environment/water_allocation_disturbance/MapServer
British Columbia	Water Rights Licences	Open (spatial)	https://catalogue.data.gov.bc.ca/dataset/water-rights-licences-public
Manitoba	Water use licence	Open (spatial)	https://web43.gov.mb.ca/Html5Viewer/Index.html?viewer=wallasExt.wallas&locale=en-US
New Brunswick	Watercourse and Wetland Alteration Permit	On demand	https://www2.gnb.ca/content/gnb/en/services/services_renderer.2935.Watercourse_and_Wetland_Alteration_Permit_.html
Newfoundland Labrador	Water rights	Open (spatial)	https://gnl.maps.arcgis.com/apps/webappviewer/index.html?id=8f9cddf172014b8d89eaa118bdfdfb40
Nova Scotia	Water withdrawal approval	On demand	https://novascotia.ca/nse/water/drinking.water.asp
Northwest Territories	Water licence	Open (document)	https://mvlwb.com/
			https://slwb.com/
			https://glwb.com/
			https://wlwb.ca/
			https://www.inuvwb.ca/register
Nunavut	Water licence	Open (document)	https://www.nwb-oen.ca/content/public-registry
Ontario	Permit to take water	Open (spatial)	https://data.ontario.ca/dataset/permit-to-take-water
Québec	Permis de prélèvement d’eau	On demand	https://www.environnement.gouv.qc.ca/eau/potable/production/
Saskatchewan	Water rights licence	On demand	https://www.wsask.ca/permits-approvals/water-allocation/
Yukon	Water licences	Open (spatial)	https://open.yukon.ca/data/datasets/water-licences-0
Canada	Investing in Indigenous community infrastructure- Water investments	Open (tabular)	https://geo.sac-isc.gc.ca/ciir-riim/ciir_riim_en.html

Note that the province of Prince Edward Island is not listed because it does not have municipal surface water licences.

**Fig. 1:**
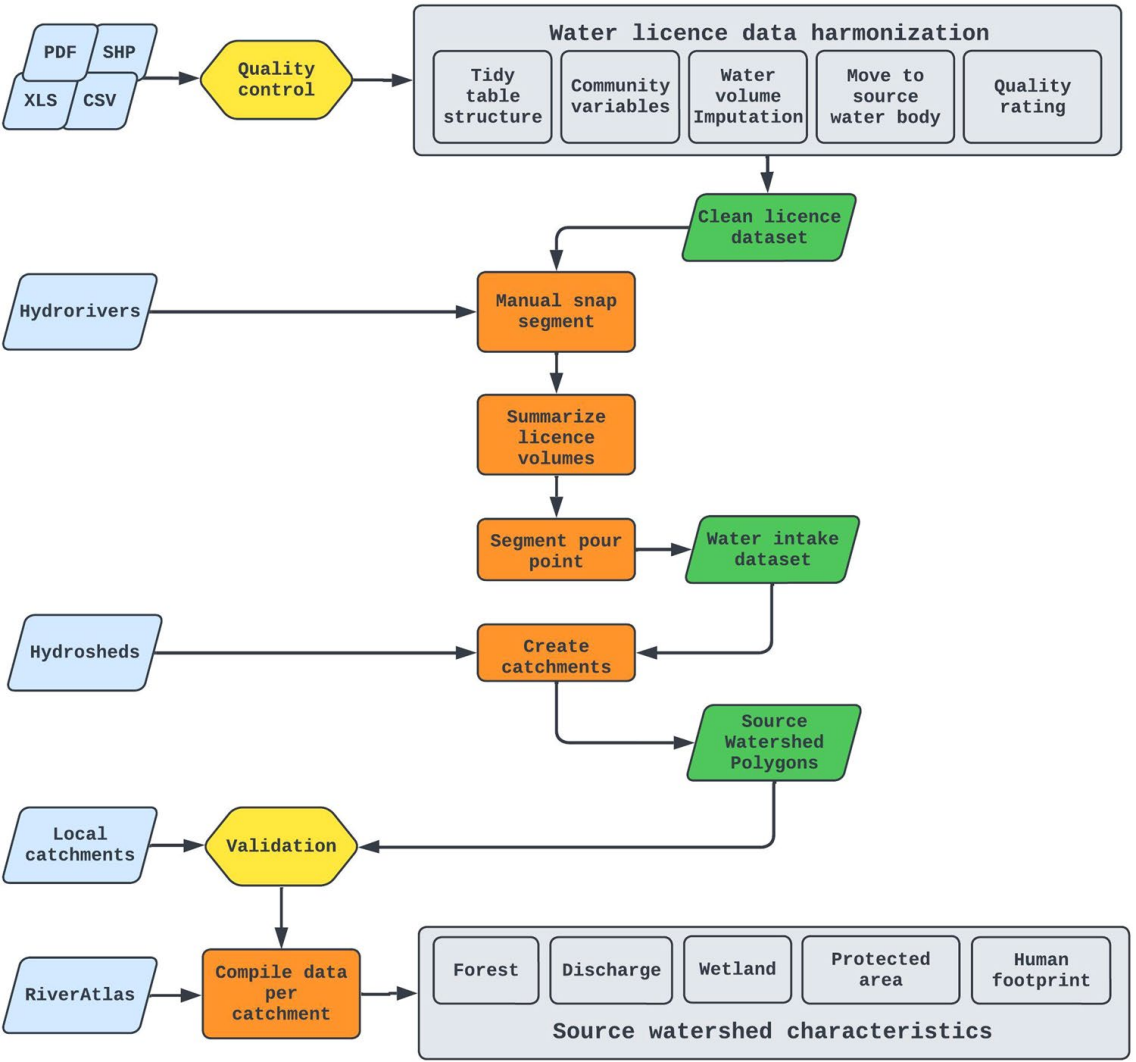
Technical steps for the creation of Can-SWaP.

We followed a four-step procedure to assemble the Can-SWaP data set. First, we gathered provincial and territorial water licence datasets that we cleaned and combined to obtain the location of municipal water licences across Canada. Second, licence points were manually snapped onto HydroRIVERS^42,43^. Third, we extracted the pourpoint of HydroRIVERS segments bearing licences to create our intake dataset. Finally, we used the intakes as outlets on the hydrologically-corrected DEM HydroSHEDS^44^ through the hydrological library WhiteboxTools^45^ to create high-quality and high-resolution source watershed polygons (Fig. 1). As expressed above, we believe that such a dataset can contribute to a national effort towards better protection of Canadian drinking water sources; its alignment with the HydroSHEDS suite of products offers a solid foundation for more data integration and analysis, including at a global scale^42,46,47^. The ever-increasing availability of open data should facilitate upgrades of this dataset by users interested in various questions related to source watershed health and protection; for instance, a number of recent datasets on land-use/land cover, wildfire severity, land tenure, and forest cover can enable temporal analysis capabilities in order to detect national trends in source watershed conditions and existing levels of protection^47–51^. The uncertainties listed herein, instead of being a deterrent for the use of the dataset, should be interpreted as targets towards the improvement of Canadian water governance; most of these uncertainties could indeed be addressed with the creation of a collaborative and standardised national dataset listing water licences and water intakes.

**Fig. 2:**
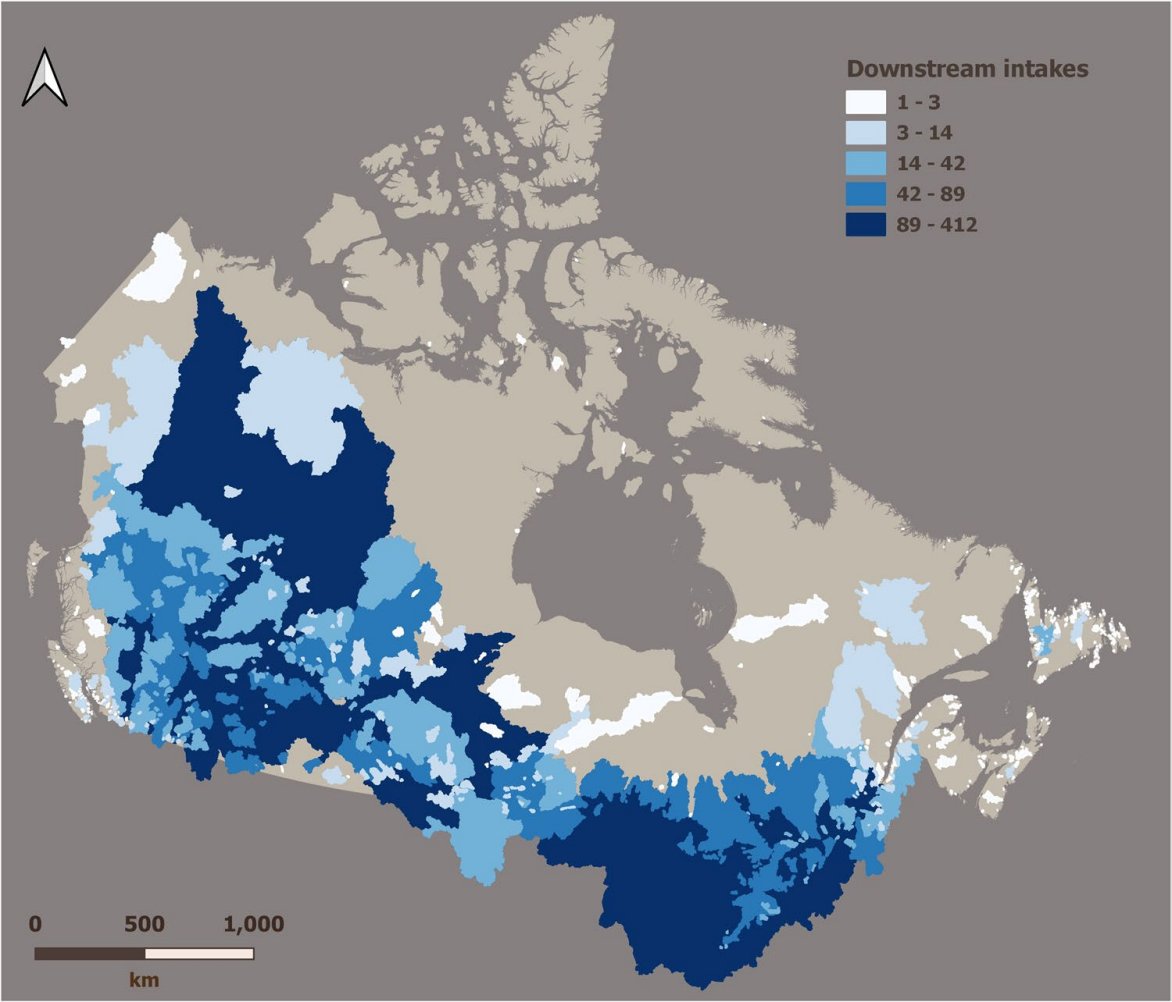
Can-SWaP coverage (n = 1574 polygons). The colour scheme shows the number of intakes a given catchment provides water to, thereby providing a high-level indicator of catchment importance for municipal water supply.

**Fig. 3:**
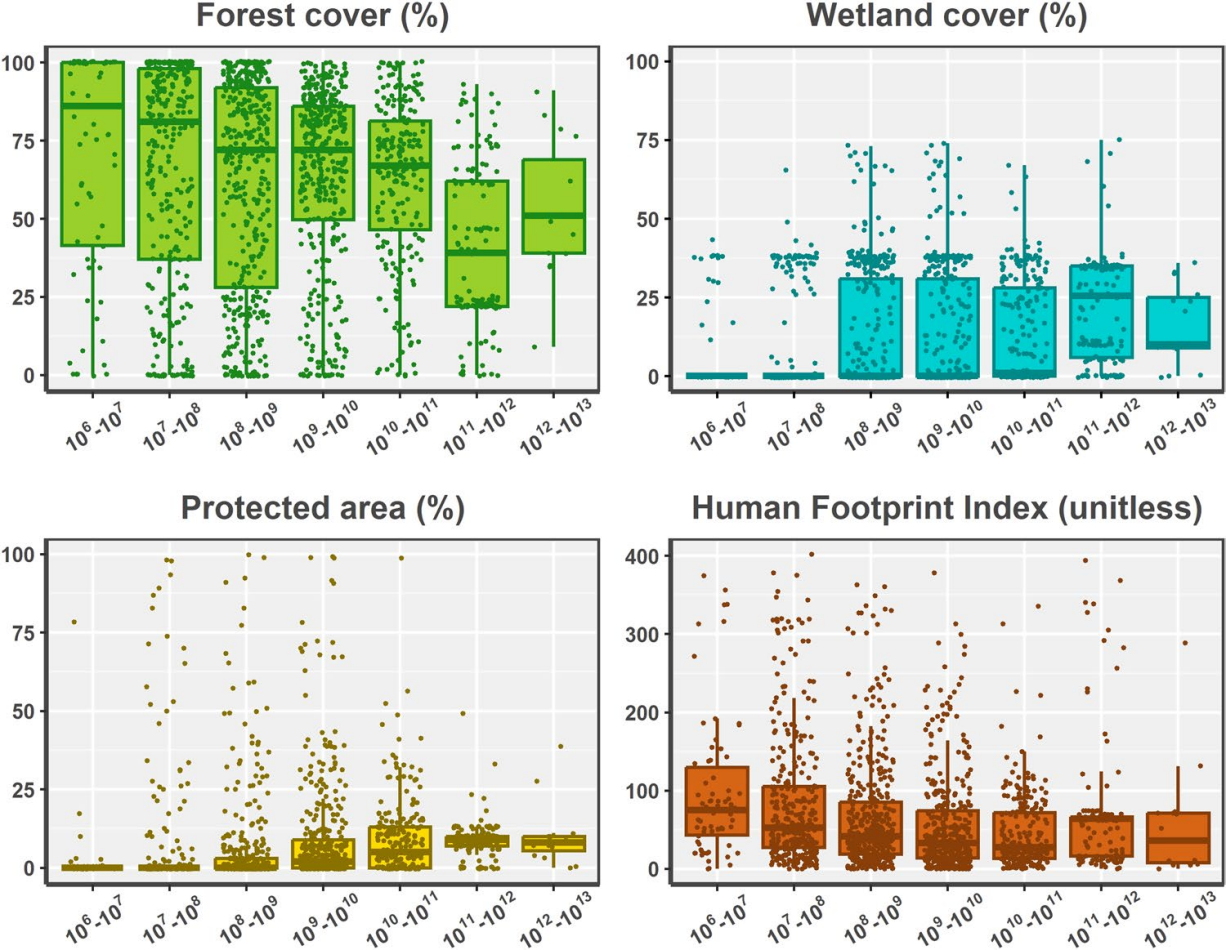
Distribution of selected environmental variables per catchment area. For all graphs, the x-axis shows classes of catchment areas on a log10 scale. For the graphs representing Forest cover, Wetland cover, and Protected area, the y-axis values are expressed in percentage (i.e., percent cover), whereas the Human Footprint Index is unitless and the y-axis displays a score equating a degree of human footprint: the higher the score, the higher the footprint.

Can-SWaP counts 1,574 polygons covering 5.3 million km², with 85% of this area located in Canada and 15% located in the United-States (i.e., lower 48 states and Alaska). Can-SWaP encompasses over 45% of the Canadian landmass and nearly 6% of the US landmass (Fig. 2). These source watersheds account for a potential maximum of 9 billion cubic metres per year of allocated water, which is nearly four times the total domestic water consumption of Canada for 2019^52^. Catchment size varies from 1.7 km² to 1,608,959 km², for a median size of 921 km². Based on information contained in RiverATLAS, we can also compute important statistics on elements of watershed conditions that have been shown to influence drinking water security (Fig. 3). For instance, each Can-SWaP catchment has an attribute “Forest Cover Extent (for_pc_use)” imported from RiverATLAS; thus, one can compute the average (i.e., an arithmetic mean) forest cover of municipal catchments, which yields 61%, thereby demonstrating the general importance of upstream forest for the protection of human health and affordability of drinking water^53–55^. Similarly, wetlands are known for their water storage and natural filtration capacities, further contributing to drinking water security^56,57^; the average wetland cover in CanSWaP is 11.9%, with nearly 60% of catchments devoid of wetlands. The role of protected areas within source catchment has also been underlined, especially for preserving water quality^58–60^; protected areas, however, only cover 7%, on average, of any Can-SWaP catchment, and only 47% of catchments had some level of protection (e.g., national or provincial park). Comparatively, catchments show an average Human Footprint Index of nearly 66, ranging from 0 to 402 (on a 0–402 scale);^61^ human footprint is a key driver of the long-term capacity of source watersheds to supply water since it accounts for anthropogenic effects on the environment^46,47^.

## Methods

We first gathered all surface water licences for provinces and territories, except for Prince Edward Island as this province does not use surface water for municipal purposes. When possible, licence data were downloaded using open-access data portals (Table 1); when not, we sent data requests out to the regional agencies responsible for water licencing. Saskatchewan and New Brunswick provided data in a spatial format, whereas Quebec and most of the Northwest Territories data were provided in a tabular format with spatial coordinates. For the northern part of the Northwest Territories (i.e., Inuvialuit) and Nunavut we accessed licence’s documentation in pdf format through their public licence registry; spatial information as well as water volumes were retrieved and used to create a spatial point layer from scratch. The 12 provincial and territorial datasets all came with very different spatial accuracy, data structures, content, and quality, making it impossible to simply merge them together (Table 2). For datasets that came in bulk (i.e., any licence types, such as industrial groundwater, with expired records), we filtered the records to keep only those that described active municipal surface licences. All data were valid up to June 2021, except for Ontario and Nova Scotia where data are only up to date until 2017.

**Table 2:** Level of information contained in provincial and territorial data useful to drinking water risk assessment.

Province/Territory	True drinking water intake (point of diversion)*	Source water body name	Community /licensee name	Allocated volume	Population serviced	Distinction primary/backup	Treatment type	Metadata
Alberta		✓	✓	✓				✓
British Columbia	✓	✓	✓	✓				✓
Manitoba	✓	✓	✓	✓				
New Brunswick	✓	✓	✓					
Newfoundland Labrador	✓	✓	✓		✓	✓	✓	✓
Nova Scotia	✓	✓	✓	✓		✓		
Northwest Territories**	✓	✓	✓	✓				
Nunavut**	✓	✓	✓	✓				
Ontario	✓	✓	✓	✓				✓
Québec	✓		✓		✓			
Saskatchewan		✓	✓	✓		✓		
Yukon	✓	✓	✓	✓				✓

Note that further information can be retrieved from other sources (see Table 1). Note that the province of Prince Edward Island is not listed because it does not have municipal surface water licences. *Location of the intake within the water body or close enough so the water source can be retrieved from the combination of record location and attributes. **Non-spatial information was retrieved from public licence registries (see Table 1).

After this first filter, each point within each regional dataset was manually checked and moved onto its surface source in QGIS based on visual analysis of Canvec 50 K^62^, Google and Bing aerial imagery and the help of Open Street Map (OSM) to identify or confirm the source. This dataset focuses on surface water supply to communities; we thus deleted records that seemed erroneous or inadequate: no apparent surface water source, private smallholder with small allocated water volumes, irrigation and other farming purposes. We added a flag when communities could clearly be identified as Indigenous (i.e., First Nations, Metis, and Inuit), either based on their name (e.g., South Indian Lake), their identification on OSM, or their census classification (e.g., Indian reserve); for northern Canada, we labelled as Indigenous all intakes within the Nunangat land—the traditional land of the Inuits^63^.

Dealing with water volumes (i.e., allocated water rights) proved more challenging. In the simplest case, one licence equates one amount of allocated water; there are many cases, however, where a community draws water from multiple locations, and thus holds multiple licences without their nominal volume recorded, and the original file shows that the total volume allocated to this same community was allocated to each licence. In such cases, it means that a summary of the total amount of water for one community that holds five licences would be five times the actual allocated volume. In similar cases, a community holds one licence but there are multiple locations water is drawn from, and simply deleting duplicates in such cases would lead to missing surface water sources in the final dataset. The simplest and most objective solution to deal with both these problems was to compute ratios: in the first case, each licence was assigned the ratio of the total volume to the number of licences (e.g., total volume = 5000; number of licences = 5; volume/licence = 1000); in the second case, each location with the same licence identifier was assigned the ratio of the total volume to the number of locations (e.g., total volume = 5000; number of locations = 5; volume/location = 1000).

Water licence data for the provinces of Québec, as well as for Newfoundland and Labrador did not contain water volumes. Instead, the population serviced by a drinking water treatment facility holding a given water licence was provided. For these two provinces, water volume allocated per licence were estimated based on the following equation for estimating residential water demand (https://legacy.winnipeg.ca/waterandwaste/dept/waterdemand.stm):

$$Average\;yearly\;water\;demand(cubic\;metres)=({215}L/capita/day+{10}{ \% })\ast {365}\ast {0.001}$$

With 215 litres being the average daily residential water use per capita in 2019 in Canada^52^. Data from New Brunswick did not contain any volume or population information, which was also the case in a few records from Newfoundland and Labrador. In those cases, we used population data from the 2016 census estimates at the community level^64^ and then used the above equation to compute yearly water volumes. The problem described in the previous paragraph was encountered on several occasions for the three above-mentioned provinces and was dealt with in the same manner (i.e., using ratios).

**Fig. 4:**
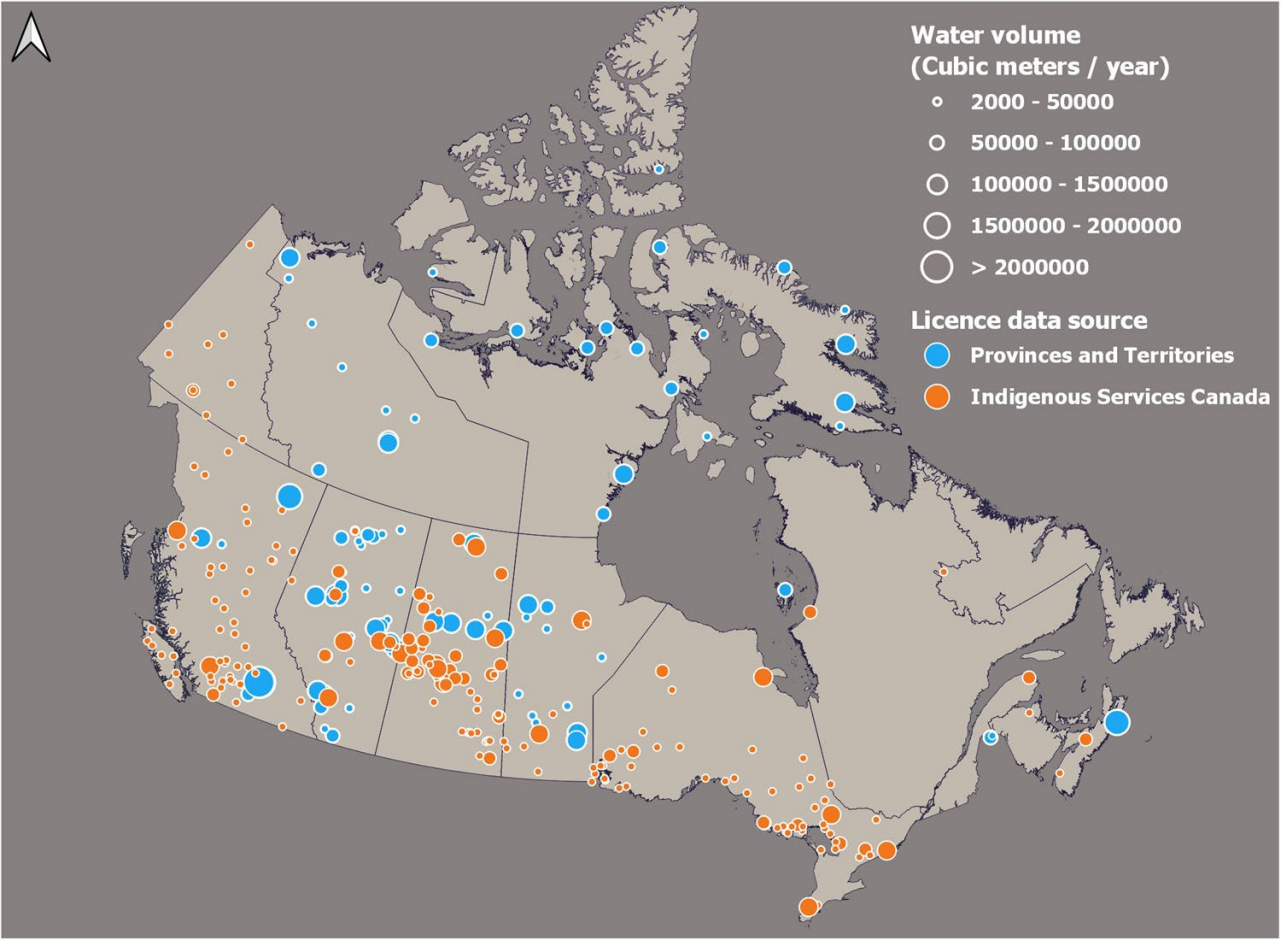
Location of Indigenous water licences from both federal (i.e., ISC) and provincial/territorial sources, after cleaning and moving them to water sources. Note the quasi-absence of Indigenous water licences in Quebec and Newfoundland and Labrador.

Indigenous water issues are of critical importance in Canada given the prevalence of water injustice in Indigenous communities resulting from a pervasive legacy of colonial water rights^9,65,66^. A preliminary assessment yielded only 113 municipal licences for Indigenous communities, while the Government of Canada’s last national assessment of First Nations water systems^67^ outlines that the figure should be closer to 235, suggesting undercounting. To avoid Indigenous being under-represented in the dataset given drinking water security struggles in their communities^8,68^, we requested information from Indigenous Services Canada (ISC, Table 1), which provided us with a listing of investments related to water supply and wastewater in the First Nations. After the removal of duplicates, the listing contained 481 records. We used the coordinates in the table to create a point layer that was then manually updated following similar steps as for provincial and territorial data. The level of detail was not sufficient to tease apart what is surface water from groundwater or wastewater; we used HydroWASTE^69^ to remove those points that spatially matched wastewater records. No information pertaining to the type of surface water resource existed in the ISC dataset; this was updated using OSM, as well as Google and Bing imagery based on the closest and most logical surface water source at a 1:50,000 scale (i.e., no salt water, no marsh, no shallow prairie pond in the middle of fields, right downstream of an existing wastewater plant). We also removed points without a nearby visible source of surface water. No water volume was provided either, and we used the population from the 2016 census information. When the community was not identified by Statcan, the population was filled in after searching in the registry of First Nations (https://fnp-ppn.aadnc-aandc.gc.ca/fnp/Main/index.aspx?lang=eng), which is only valid up to 2016. Several communities did not have an official recorded population and were then deleted. This aggressive cleaning method was necessary since a community can receive funding for both drinking water and wastewater. Our final ISC data, containing 219 records, is therefore on the conservative side. We compared the resulting point layer with the 113 existing licences to check for potential duplicates; none were found (Fig. 4).

The thirteen separate clean licence files (12 for provinces and territories, 1 for the federal) were then merged into one spatial point dataset containing 3,341 records. These points were manually snapped to HydroRIVERS in QGIS so watersheds could be traced automatically using the hydrologically-corrected elevation model HydroSHEDS, and various river attributes could be retrieved from RiverATLAS^42,44^. Besides the benefit of using a well-documented and robust suite of hydrographic products, RiverATLAS provides numerous spatial variables of relevance to source-watershed studies that were kept in the final product: annual discharge, upstream forest cover, upstream wetland cover, upstream protected area, and upstream human footprint. Differences in precision between the OSM-imagery combination and HydroRIVERS prevented us from using an automated snap function; a test indeed showed that this process introduced a large amount of attribution errors. In cases where a licence could not be matched with a river segment within the same catchment inside a two-kilometre radius, the licence point was discarded; we made this decision given the uncertainty in HydroSHEDS and the HydroRIVERS network for small reaches and catchments, especially at higher latitudes and in flatter areas^42,44^. A total of 259 licences (7.8%) were discarded at this step. All HydroRIVERS segments bearing a licence point were then converted to their pourpoint, using the function ‘Extract specific vertices’ in QGIS; the vertex index was −1, meaning the last point of a segment. The resulting water intake dataset contained 1594 points to be used as outlets for the creation of source watersheds. We used the function ‘unnest_basins’ in the R library WhiteboxTools (https://github.com/opengeos/whiteboxR)^45^ to create a layer of individual catchments: for a given outlet, this function delineates the whole watershed irrespective of any other upstream outlet, resulting in overlaps with catchments located upstream, while many GIS watershed tools will create nested basins by default, i.e., non-overlapping catchment polygons only accounting for the area located between two hydrologically-connected outlets. Using unnested catchments gives the ability for managers to compile a “full picture” of the state of the watershed by capturing the entirety of the processes happening upstream that might influence, or even compromise raw water. The raster files resulting from this ‘unnest_basins’ function were converted to polygons using the ‘raster_to_vector_polygons’ function in the same library and merged into one single layer (see R code on Github). The final polygon layer counted 1574 catchments, or 20 less than expected based on the number of pourpoints; this is explained by the existence of 20 confluences, i.e., spatial point duplicates. Given that these points do not influence the boundaries of the final polygon layer, they were exported as their own spatial layer containing allocated water volumes and made available to users in the public data repository. Finally, because of the built-in uncertainty from using ISC data, we provide an intake file without ISC licences, for which allocated water volumes per intake were updated accordingly; this file will be useful for users wanting to generate a version of Can-SWaP with provincial and territorial licence information only.

Each catchment shared a unique identifier with the intake dataset (‘fid’ in QGis); this one-to-one relation was used to retrieve information from RiverATLAS and generate the final version of the product. We kept the following attributes: annual average natural discharge, upstream forest cover extent, upstream protected area extent, total upstream wetland extent, and upstream 2009 human footprint (https://www.hydrosheds.org/hydroatlas)^42^. Natural discharge was updated to represent the average total discharge in a year instead of the average discharge per second. Field names were updated as well (i.e., upper case).

## Data Records

The national water licence dataset created and used to locate intakes for building source catchments cannot be shared due to usage restrictions coming with several provincial and territorial data sources. We invite interested readers to access open datasets or to address data requests through the web pages of Provinces and Territories listed in Table 1.

The final datasets (Table 3) with metadata are provided in the open format geopackage (.gpkg): HydroRIVERS pourpoints, including ISC data; HydroRIVERS pourpoints, without ISC data; catchments based on those pourpoints, including ISC data (i.e., Can-SWaP); HydroRIVERS confluence pourpoints (i.e., duplicates, *n = *20). Also provided is a ‘README.txt’ file providing basic information on the layers and describing the attributes available in the dataset (i.e., name and content of the fields). Material can be downloaded from the Figshare repository associated with this publication (“Can_SWaP_V1.zip”)^70^. The original water licence data were active as per June 2021, except for Ontario and Nova Scotia where records only extended up to 2017. The data available in the repository is projected in EPSG:3979 Canada Atlas Lambert, except for the two intake files in EPSG:4326 WGS84 for ease of use when creating catchments with HydroSHEDS. In the latter case, the user will have to convert both files to ESRI shapefiles to ensure compatibility with WhiteboxTools; users must be aware of possible data quality loss when doing so.

**Table 3:** Files available within the Can-SWaP Figshare repository.

Filename	Data type	Content
Intakes_HydroRivers_Segments_With_Licence.gpkp	Point	Water intakes (pourpoints) based on the location of all licences
Intakes_HydroRivers_Segments_With_Licence_WGS84.gpkg	Point	Water intakes (pourpoints) based on the location of all licences (WGS84)
Intakes_HydroRivers_Segments_With_Licence_NoISC.gpkg	Point	Water intakes based on the location of licences excluding ISC ones
Intakes_HydroRivers_Segments_With_Licence_NoISC_WGS84.gpk	Point	Water intakes based on the location of licences excluding ISC ones (WGS84)
Intakes_HydroRivers_Segments_With_Licence_Confluence.gpkg	Point	Duplicated intakes (pourpoints) at confluences
Can-SWaP_AllLicences.gpkg	Polygon	Can-SWaP based on the location of all water intakes
Can-SWaP_AllLicences.csv	Table	Can-SWaP based on the location of all water intakes (non-spatial)
Can-SWaP_Readme.txt	Text	High-level description of folder content and files

## Technical Validation

The quality of Can-SWaP was assessed against available provincial datasets of municipal catchments, individual catchment boundaries from Water Survey of Canada, and maps of municipal catchments published in government reports (Table 4). British Columbia, Newfoundland and Labrador, and New Brunswick provide open-access layers of their source watersheds. Interestingly, these regional datasets contain additional information relevant to SWP that are not in the licence files, such as the type of water treatment or the status of the catchment as primary or backup source. These layers were used to compare the number of catchments, their average area, and their degree of overlap (Table 5). For Newfoundland and Labrador and British Columbia, we first sorted protected water supplies to only keep areas providing surface water. Given those datasets and maps come from official sources, we assumed that the information they contain represented the “true” topographic catchments that Can-SWaP would be assessed against.

**Table 4:** List of material and type of assessment that was conducted to validate Can-SWaP.

Reference name	Reference type	Source	Link	Type of assessment
Community Watersheds - Current	Spatial polygon layer	Government of British Columbia	https://catalogue.data.gov.bc.ca/dataset/community-watersheds-current	Spatial overlap
Protected watersheds	Spatial polygon layer	Government of New Brunswick	https://gnb.socrata.com/Geographic-Data/Protected-Watersheds-Bassins-versants-prot-g-s/uuey-4g2f	Spatial overlap
Public water supplies	Spatial polygon layer	Government of Newfoundland and Labrador	https://www.gov.nl.ca/ecc/waterres/gis/gis/	Spatial overlap
Edmonton (North Saskatchewan River)	Spatial polygon layer	Water Survey of Canada (gauge 05DF001)	https://collaboration.cmc.ec.gc.ca/cmc/hydrometrics/www/HydrometricNetworkBasinPolygons/	Spatial overlap
Calgary (Elbow river)	Spatial polygon layer	Water Survey of Canada (gauge 05BJ008)	https://collaboration.cmc.ec.gc.ca/cmc/hydrometrics/www/HydrometricNetworkBasinPolygons/	Spatial overlap
Prince Albert (North Saskatchewan River)	Spatial polygon layer	Water Survey of Canada (gauge 05GG001)	https://collaboration.cmc.ec.gc.ca/cmc/hydrometrics/www/HydrometricNetworkBasinPolygons/	Spatial overlap
Timmins (Mattagami River)	Spatial polygon layer	Water Survey of Canada (gauge 04LA001)	https://collaboration.cmc.ec.gc.ca/cmc/hydrometrics/www/HydrometricNetworkBasinPolygons/	Spatial overlap
Stellarton (East River)	Spatial polygon layer	Water Survey of Canada (gauge 01DP003)	https://collaboration.cmc.ec.gc.ca/cmc/hydrometrics/www/HydrometricNetworkBasinPolygons/	Spatial overlap
Sable à Saguenay (Rivière aux Sable)	Spatial polygon layer	Water Survey of Canada (gauge 02RH072)	https://collaboration.cmc.ec.gc.ca/cmc/hydrometrics/www/HydrometricNetworkBasinPolygons/	Spatial overlap
Portrait des bassins versants des prises d’eau municipales de surface	Technical report	Gouvernement du Québec	https://cmquebec.qc.ca/wp-content/uploads/2020/07/2020-07_Portrait_BVPE-CMQ_vf-WEB.pdf	Visual

**Table 5:** Quality assessment of Can-SWaP against available provincial source catchments datasets.

Province	Number of source catchments	Number of catchments in Can-SWaP	Mean (range) size of source catchments (km^2^)	Mean (range) size of source catchments in Can-SWap (km^2^)	Mean (range) overlap Province by Can-SWaP (%)	Mean (range) overlap Can-SWaP by Province (%)
British Columbia	337	440	30 (0.02–716)	14,552 (1.7–212,227)	84 (0–100)	21 (0–100)
New Brunswick	30	25	68 (2–371)	3,775 (7–20,142)	72 (0–100)	31 (0–91)
Newfoundland and Labrador	283	202	42 (0.1–4,252)	561 (4–68,838)	44 (0–100)	38 (0–98)

**Fig. 5:**
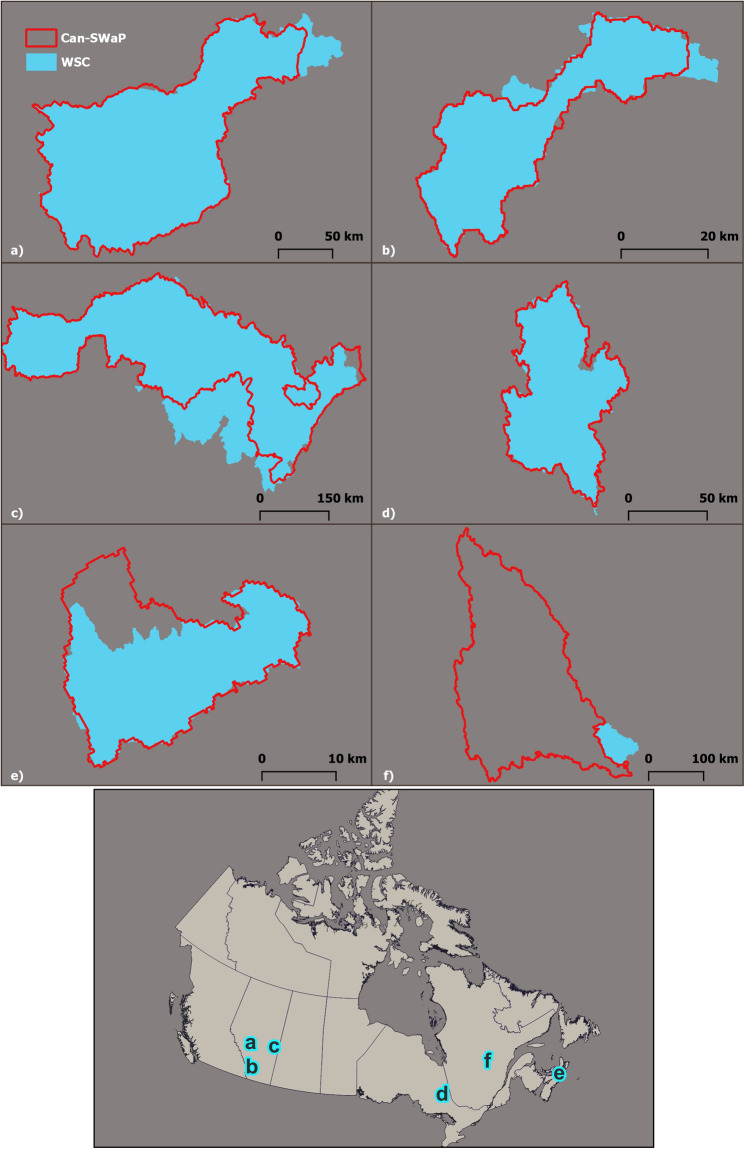
One-to-one differences between Can-SWaP and WSC catchments for: (**a**) Edmonton (Alberta), (**b**) Calgary (Glenmore reservoir, Alberta)), (**c**) Prince Albert (Saskatchewan), (**d**) Timmins (Ontario), (**e**) Stellarton (Nova Scotia), (**f**) Saguenay (Québec). The map at the bottom shows the general location of these catchments in Canada.

Across the country, several cities and towns sitting on major waterways often have a gauge that measures water flow maintained by government agencies; we retrieved these catchments from historical hydrographic records and the corresponding catchment layer maintained by the Water Survey of Canada. For these catchments (Fig. 5), we compared area and level of overlap (Table 6). Finally, Québec City provides an online document with a general presentation of their source catchments and we visually inspected their map for comparison with our dataset.

**Table 6:** Quality assessment of Can-SWaP against gauged catchments from Water Survey of Canada (WSC).

Community (Stream)	Size WSC catchment (km²)	Size Can-SWap catchment (km²)	Overlap WSC by Can-SWaP (%)
Edmonton (North Saskatchewan River)	28,109	26,325	95
Calgary (Elbow river)	1,225	1,112	90
Prince Albert (North Saskatchewan River)	153,295	130,467	81
Timmins (Mattagami River)	6,179	6,301	98
Stellarton (East River)	412	535	97
Sable à Saguenay (Rivière aux Sable)	3,400	73,389	0.7

**Fig. 6:**
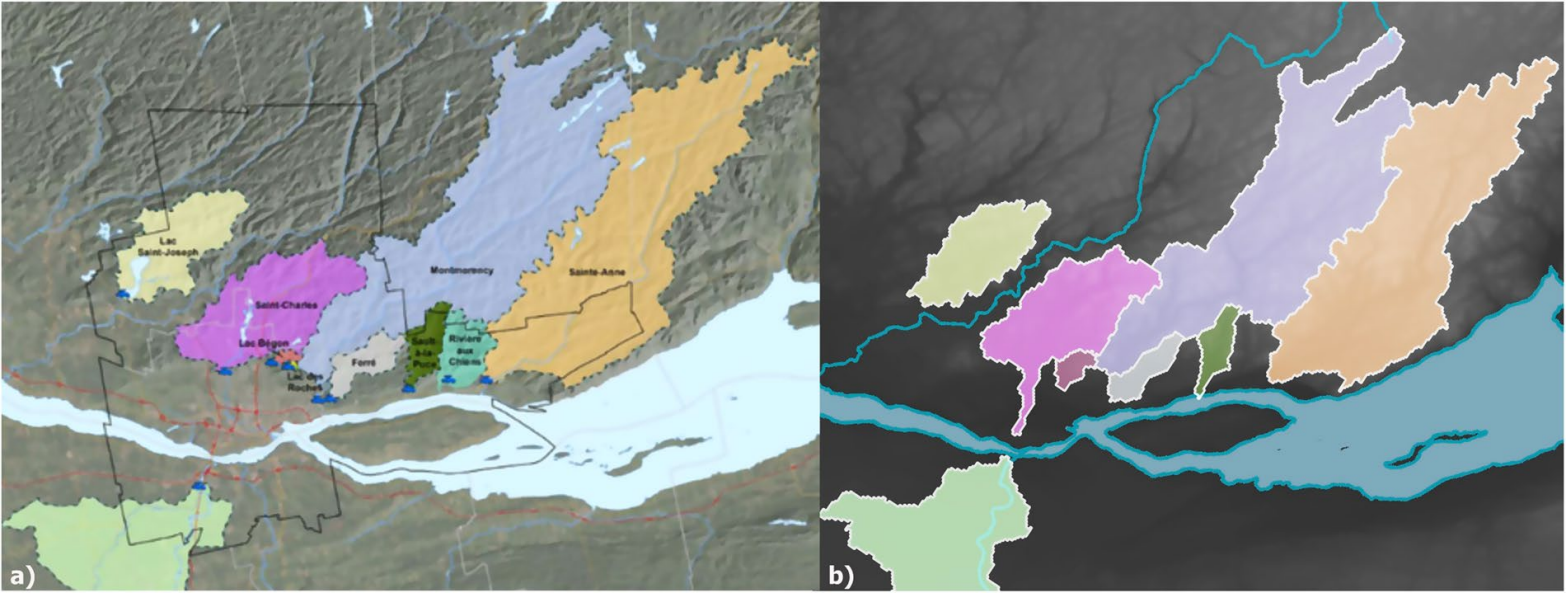
(**a**) Source watersheds for the city of Québec as published in official documentation; (**b**) Source watersheds for the city of Québec in Can-SWaP. There is a good general correspondence between both data sources, although it seems that Can-SWaP catchments are slightly larger, which could be explained by the rather coarse resolution of HydroSHEDS (if one assumes that regional authorities use the highest resolution available for tracing catchments).

Based on comparison with provincial catchment datasets (Table 5) using the ‘overlap analysis’ tool in QGIS, catchment size tends to be overestimated by Can-SWaP, while the total number of catchments is relatively similar. These overestimations negatively affect the level of overlap, although some catchments match well (up to 98% overlap in Newfoundland and Labrador). The catchment-to-catchment comparison between Can-SWaP and WSC yields generally good results (Fig. 5, Table 6), although the catchment for Sable à Saguenay is completely off the mark; the “true” topographic catchment is, however, captured by an intake located a few kilometres downstream for the community of Saguenay (Québec). Similarly, a comparison with source catchments for Québec City suggests an acceptable level of accuracy (Fig. 6).

## Usage Notes

### Potential applications

Even though Can-SWaP was originally designed with source water protection in mind, one can imagine further applications of the dataset, such as risk analysis of municipal water shortage given climate extremes and changes to consumption patterns^71^, the study of interbasin transfers^72^, the understanding of mass balance inequalities^73^, or the in-depth analysis of historical forest disturbances (e.g., wildfires^74^) or of industrial accidents (e.g., oil spill^75^) effects on water supply. Doing so would require the dataset to be further developed so it integrates meteorological and catchment attributes that would enable its use in large-sample studies, similar to other catchment datasets such as CAMELS and HYSETS^76,77^.

### Uncertainties

The first source of uncertainty pertains to the water licence files: they all come in a diversity of formats, with variable quality and content. The licences are also not always up to date, and the information they contain can be hard to interpret, even when metadata is provided. Three of these files did not provide water volume information, and several other files failed at recording actual allocated water amounts per intake, leading to over-estimations of water rights if the user is not careful. None of them provided an ideal structure with an optimal amount of information that could be leveraged; hence, despite a thorough effort to produce a harmonised dataset to work with, our national licence file necessarily carries over these uncertainties. In particular, water volumes in Can-SWaP must be used with caution, for several reasons: they represent a potential maximum water use, not the actual withdrawal; where ratios were used, they can be over- or under-estimated; 31% of licences had their volume derived from population data, likely leading to estimation errors; they can be associated to a backup catchment and thus only used occasionally. Another consequence of using licence files also means that Can-SWaP likely overestimates the number of source watersheds; indeed, it was often not possible to separate licences for community drinking water supply from other municipal water uses. Nonetheless, using municipal licences remains the best proxy to drinking water demand in the Canadian context, and the quality control shows that all source areas are captured by Can-SWaP regardless of these challenges.

A second source of uncertainty relates to the impossibility to automate most of the cleaning and harmonisation steps, and this dataset is primarily the product of manual labour with updates performed on a case-by-case basis; it would therefore be impossible to describe each of them in greater detail. It should be noted that several recent datasets published in high profile journals also have a fair amount of manual work involved^78,79^. Despite great care, it is possible that users will find errors, which should be reported to the authors for corrections in future updates. Nevertheless, the location of water intakes (i.e., HydroRIVERS pourpoints) derived from the location of licences must not be interpreted as their true location. Here again, this relates to the original licence files in which the quality of spatial information is highly variable, but it also relates to the limitations of HydroRIVERS and HydroSHEDS with respect to the DEM resolution and connectivity errors in the river network, which makes it hard to capture small catchments. The overestimation of catchment size in Can-SWaP is one likely consequence. Further, 259 licence points did not match HydroRIVERS, especially in Newfoundland and Labrador and parts of British-Columbia where topography is complex and communities rely on many small source catchments, and in areas of gentle topography (e.g., the floodplain of the St Lawrence River). The upcoming version of HydroSHEDS —HydroSHEDS V2 (https://www.hydrosheds.org/hydrosheds-v2) —as well as other efforts to produce high-resolution continental and global hydrographic products will surely help produce improved versions of Can-SWaP in the years to come^80–82^.

Technical validation shows important differences between provincial source catchment datasets; notwithstanding the various quality issues in the original licence datasets, it could also suggest that current source catchment boundaries are erroneous and at risk of having inadequate levels of protection. It is also possible that some of the reference catchments used for the validation capture areas where groundwater inputs to surface water supply are substantial; however, groundwater catchments can be misaligned with surface catchments defined by topographic features, leading to spatial discrepancies and thus protection deficit^73,83^.

The level of uncertainty with records related to Indigenous communities is high. It is unclear why provincial and federal records do not match regarding the number of water licences and water treatment facilities. Generally speaking, Canadian water law as it relates to Indigenous water rights is a complex matter, and the above-mentioned uncertainty is most likely a consequence of this complexity^84^. It is also possible that some Indigenous communities shifted to intercommunal water sharing agreement and are now served by a nearby water treatment plant from a non-Indigenous community^85^. Notwithstanding these serious quality issues, integrating this data was deemed paramount given the long-lasting water insecurity that have existed in Indigenous communities; in other words, in this context, low-quality data is better than no data at all.

We want to stress that this dataset is ideally meant for national and international comparisons (see for e.g.^46^,) and that addressing specific socio-environmental issues is better done with a focus on a single watershed, with ad-hoc methods and, when necessary, datasets^86^. This dataset therefore does not integrate all the possible hydrographic, social, and environmental descriptors that might be relevant for a particular location or issue to be investigated, and it is not meant to be.

## Data Availability

The code used to create Can-SWaP (i.e., generating the watersheds, populating the attributes with RiverATLAS, extracting statistics) is available on Github at https://github.com/FNRobinne/Can-SWaP.git, and as an archive on Figshare^87^. It will require the user to download the necessary HydroSHEDS products. Running the code requires RTools installed on the user’s machine. The final versions of these datasets were built in QGis 3.28.3-Firenze, R v4.2.2, RStudio 2022.12.0 Build 353, and ArcGIS 10.x for some data conversion from ESRI proprietary formats into OGC formats.
